# Formation of 5-Hydroxy-3-methoxy-1,4-naphthoquinone and 8-Hydroxy-4-methoxy-1,2-naphthoquinone from Juglone

**DOI:** 10.5402/2012/274980

**Published:** 2012-09-23

**Authors:** Bastian Blauenburg, Mikko Metsä-Ketelä, Karel D. Klika

**Affiliations:** ^1^Department of Biochemistry and Food Chemistry, University of Turku, Vatselankatu 2, 20014 Turku, Finland; ^2^Department of Chemistry, University of Turku, Vatselankatu 2, 20014 Turku, Finland

## Abstract

From the treatment of 5-hydroxy-1,4-naphthoquinone (juglone) with acetic anhydride and H_2_SO_4_ followed subsequently by treatment with methanolic HCl, 5-hydroxy-3-methoxy-1,4-naphthoquinone (3-methoxy juglone) and 8-hydroxy-4-methoxy-1,2-naphthoquinone were obtained as products rather than the anticipated product 2,5-dihydroxy-1,4-naphthoquinone (2-hydroxy juglone). The reaction and the identification of the products are discussed in terms of NMR and DFT calculations.

## 1. Introduction

**Scheme 1:**
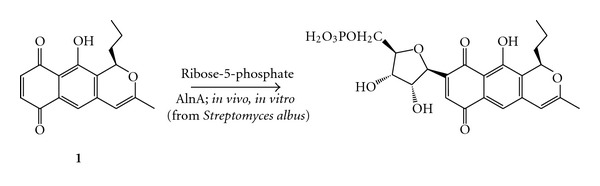
The C-ribosylation of (*R*)-prealnumycin (**1**) by *Streptomyces albus*.

As part of our ongoing investigation [1] into the C-ribosylation of (*R*)-prealnumycin (**1**) by AlnA, an enzyme produced by *Streptomyces albus* (Scheme 1), it was decided to test various 1,4-naphthoquinone substrates against AlnA in order to identify the structural motifs necessary for, or inhibitory to, reaction.

**Figure 1:**
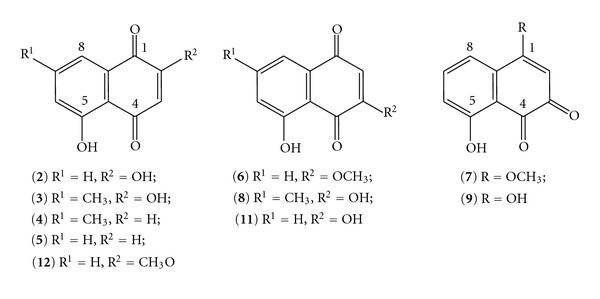
The structures of the 5-hydroxy-1,4-naphthoquinones (juglones) and 8-hydroxy-1,2-naphthoquinones discussed in this work. Nb. For ease of comparison, the atomic numbering of the juglone series is maintained for **7** and **9** in discussion in the text even though it is unconventional.

Thus, we considered 2,5-dihydroxy-1,4-naphthoquinone (**2**, 2-hydroxy juglone, Figure 1) as a test substrate since it could potentially reveal aspects of the charge required to be present on C-3 for the reaction to proceed. Additionally, the facile preparation of 2,5-dihydroxy-7-methyl-1,4-naphthoquinone (2-hydroxy-7-methyl juglone, **3**) from 5-hydroxy-7-methyl-1,4-naphthoquinone (7-methyl juglone, **4**) had been reported [2] by treatment first with acetic anhydride and H_2_SO_4_ and then subsequently with methanolic HCl. After treating 5-hydroxy-1,4-naphthoquinone (**5**, juglone) as prescribed, we obtained not 2-hydroxy juglone (**2**) as anticipated, but rather 5-hydroxy-3-methoxy-1,4-naphthoquinone (**6**, 3-methoxy juglone) and 8-hydroxy-4-methoxy-1,2-naphthoquinone (**7**). Mahapatra et al. [2], in turn, seem to have taken their preparation from Lillie and Musgrave [3] who performed precisely the same reaction. Interestingly, whilst the former workers only reported the one product, namely, 2-hydroxy-7-methyl juglone (**3**), the latter pair reported the formation of both **3** (as the major product) and its regioisomer 3,5-dihydroxy-7-methyl-1,4-naphthoquinone (3-hydroxy-7-methyl juglone, **8**). Mahapatra et al. [2] only reported ^1^H NMR chemical shifts (*δ*_H_) for their compound, and indeed only one OH signal—which they assigned as HO-5—with no carbon or correlation spectra reported. As best we can ascertain, it appears that Mahapatra et al. [2] based their structural assignment on the work of Lillie and Musgrave [3] and the *δ*_H_ of HO-5 (vide infra). This structural assignment therefore carries with it some concern, not because it is a labile proton *per se*, but rather because of its value in relation to the reported value [3]. The correctness of this structural assignment is scrutinized in this work. 

Both 3-methoxy juglone (**6**) and 8-hydroxy-4-methoxy-1,2-naphthoquinone (**7**) are known compounds, **6** in particular has been studied well synthetically [4, 5], is a widely spread naturally occurring compound present, for example, in a number of species of the genus *Juglans* [6–9], and possesses significant biological activity [7–9]. Compound **7** is unknown outside the laboratory [4, 10, 11], and overall there are only limited reports on it, though it too has potential biological activity of significance [10]. Herein we report on the synthesis of these two compounds and discuss the reaction and identification of the products in terms of NMR and DFT calculations. 

## 2. Results and Discussion

Our original intent was to follow analogously the preparation of Mahapatra et al. [2] who reported the synthesis of what they considered to be 2-hydroxy-7-methyl juglone (**3**) from 7-methyl juglone (**4**) and apply the same conditions to juglone (**5**) in order to obtain 2-hydroxy juglone (**2**). After performing the synthetic preparation, we isolated two major components in comparable amounts and readily identified them as 3-methoxy juglone (**6**) and 8-hydroxy-4-methoxy-1,2-naphthoquinone (**7**). Whilst fully expecting the first eluting compound from column chromatography over silica gel to be 2-hydroxy juglone (**2**) rather than 3-methoxy juglone (**6**), it was immediately evident that a methoxy group was present in the molecule by inspection of the ^1^H NMR spectrum (see Table 1), and furthermore, its general location was evident from the long-range coupling and an NOE to a vinyl-type proton. Although the methoxy group was then expected to be attached at C-2, the correlation of the vinyl-type proton (H-2) to C-8a in the HMBC spectrum was categorical in placing it at C-3 in addition to an NOE between the methoxy group protons and HO-5. Following these pivotal connectivities, the rest of the NMR analysis was consistent with the assigned structure. Turning our intention to the second isolate **7** eluting from the column, it exhibited some strongly divergent NMR spectral differences to **6** (e.g., *δ*_C3_, *δ*_C1_, etc.), and the reason quickly became apparent as this isomer of **6** was not a 1,4-quinoid structure, but rather a 1,2-quinoid structure. Notable correlations identifying this compound as 8-hydroxy-4-methoxy-1,2-naphthoquinone (**7**) were H-2 to C-8a and H-8 to C-1 (conspicuous by its *δ*_C_ of 168.15 ppm) in the HMBC spectrum and the NOE between the methoxy group and H-8.

**Scheme 2:**
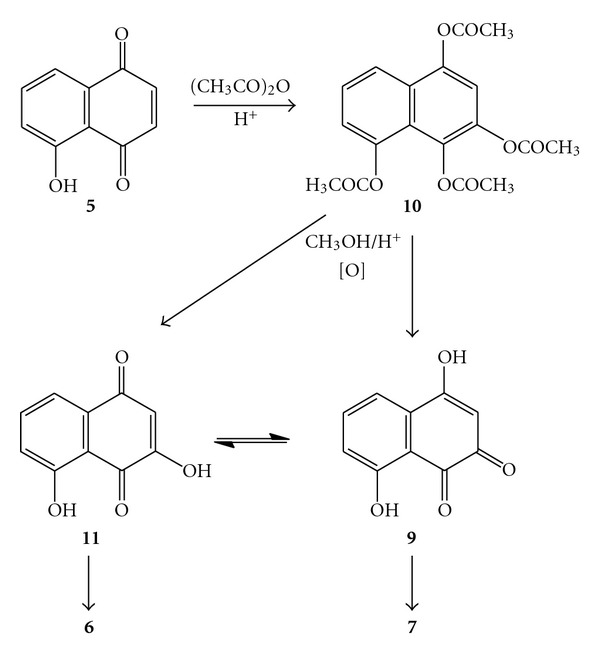
The reaction sequence from juglone (**5**) leading to the isomeric 3-methoxy juglone (**6**) and 8-hydroxy-4-methoxy-1,2-naphthoquinone (**7**).

Though the obtainment of the methoxy derivatives 3-methoxy juglone (**6**) and 8-hydroxy-4-methoxy-1,2-naphthoquinone (**7**) was unexpected, the presence of methoxy groups may be rationalized simply by methylation of the freed hydroxyls after hydrolysis (Scheme 2). The surprising occurrence of **7** can be accounted for by either of the following: if 4,8-dihydroxy-1,2-naphthoquinone (**9**) is not produced directly during the hydrolytic deacetylation concomitant with spontaneous oxidation of the tetraacetylated intermediate **10**, then it arises through prototropic tautomerism from 3,5-dihydroxy-1,4-naphthoquinone (3-hydroxy juglone, **11**) with which it is in equilibrium with in solution [4]. This has been demonstrated by obtaining **7** as part of a mixture together with **6** when 2-hydroxy juglone (**2**) is methylated [4]. Given the results of our reaction together with those of Mahapatra et al. [2] and Lillie and Musgrave [3], it seems that despite the deceptive simplicity of the reaction, it can be highly variable in terms of regiospecificity since sometimes attack at C-2 dominates [3], sometimes attack occurs exclusively at C-3 (this work), or sometimes attack seemingly occurs exclusively at C-2 [2] (vide infra).

To first address the question of regiospecificity, DFT calculations revealed that 2-hydroxy juglone (**2**) is 1.40 kcal mol^−1^ more stable than 3-hydroxy juglone (**11**), similar results were obtained for 2-hydroxy-7-methyl juglone (**3**, 0.00 kcal mol^−1^) and 3-hydroxy-7-methyl juglone (**8**, 1.24 kcal mol^−1^) in consideration of the work of Mahapatra et al. [2] and Lillie and Musgrave [3], and furthermore, similar results were also obtained for 2-methoxy juglone (**12**, 0.00 kcal mol^−1^) and 3-methoxy juglone (**6**, 0.66 kcal mol^−1^) in consideration of the methoxy derivatives isolated in this study. Thus, in each case, the 2-substituted derivative was calculated to be the thermodynamic product, which is consistent with the results of Mahapatra et al. [2] and Lillie and Musgrave [3] but does not account for the observations herein. To consider if the C-2 position was more susceptible to nucleophilic attack, the atomic charges on C-2 and C-3 were examined: in juglone (**5**), the Mulliken charge on C-2, *Q*_C2_, was calculated to be −0.182 cf. *Q*_C3_ at −0.176. These differences did not differ appreciably for 7-methyl juglone (**4**) where charges of −0.184 and −0.175, respectively, were found for *Q*_C2_ and *Q*_C3_. These charge differences are considered inconsequential to the course of the reaction, either between 2- and 3-substituted products or for differences observed between 7-methyl juglone (**4**) and juglone (**5**).

**Scheme 3:**
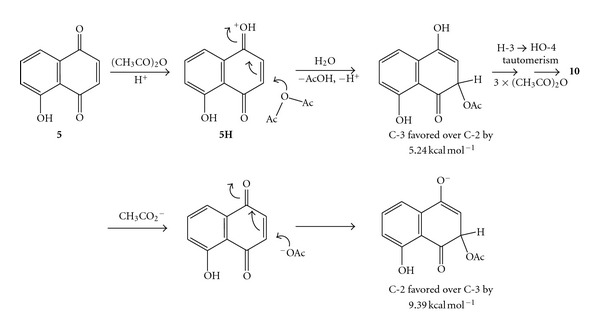
Intermediates resulting from attack by acetic anhydride/H^+^ or acetate ion.

Kinetically however, it would appear that, based on the energetic preference by 5.24 kcal mol^−1^ for the intermediate in the case of attack by acetic anhydride on protonated juglone (**5H**) at the C-3 position over the C-2 position (Scheme 3), 3-hydroxy juglone (**11**) would be the preferred product after oxidative hydrolysis. Interestingly, attack by acetate to yield the enolate ion provides the opposite effect, and then some with attack at the C-2 position preferred over the C-3 position by 9.39 kcal mol^−1^; that is, 2-hydroxy juglone (**2**) would be expected as the preferred end-product. Since the reaction was conducted under acid-catalyzed conditions and is likely to be irreversible, it is concluded that 3-hydroxy juglone (**11**) should be the dominant product, essentially in concert with observations in this work since 3-substituted products (**6** and **7**) were obtained. Kinetically one can anticipate similar results for 7-methyl juglone (**4**) as for the reaction of juglone (**5**). However it is clear that there must be a fine balance between the competing pathways, and even slight perturbation of the conditions could lead to very different results being obtained.

To gain further insight into these structures, DFT calculations of their ^1^H and ^13^C NMR chemical shifts (*δ*_H_s and *δ*_C_s, resp.) were performed (see Table 2) as these have proven to be of considerable assistance in structural analysis [14–16]. Interestingly, there is nothing in the calculated *δ*_H_s of the nonlabile protons in 2-hydroxy juglone (**2**) and 3-hydroxy juglone (**11**) to categorically distinguish between the two structures. The same holds for the calculated *δ*_H_s of the nonlabile protons in 2-hydroxy-7-methyl juglone (**3**) and 3-hydroxy-7-methyl juglone (**8**) and for the methoxy derivatives 2-methoxy juglone (**12**) and 3-methoxy juglone (**6**), with the latter pair even more similar. Thus, to reliably distinguish between the two compounds in any of these pairs based on the *δ*_H_s of the nonlabile protons, one must be in possession of both compounds. The exception is for the exchangeable HO-5 protons where significant differences are clearly evident. Usually though, labile protons are considered unreliable for assignment purposes based on experimental *δ*_H_ however, the experimental *δ*_H_ of OH protons can, if certain conditions are met, namely conditions conducive to slow exchange of the protons, be treated as reliable for assignment purposes [3]. With respect to calculated *δ*_H_s, although the chemical shifts of labile protons are generally problematic to calculate in absolute terms, they are normally reliably found in their correct chemical shift order. When exchangeable protons are strongly hydrogen bonded though, they can be accurately calculated, and this is clearly evident in the case of **2** and **11** (experimental values of 12.28 ppm and 11.04 ppm, resp. [3]) and for **6**. Thus, HO-5 is the only proton with a decisive indication of isomer identification in these pairs of compounds. 

Mahapatra et al. [2] only observed one OH signal, assigned as the HO-5 proton, in which case the other OH signal must therefore be broad due to exchange despite the possibility of it too being intramolecularly hydrogen bonded. Therefore, under such conditions the *δ*_H_ reliability of the observed OH proton must be treated with caution. However, one inference can be made: the reported [2] value for *δ*_HO5_, 11.69 ppm, is intermediate between *δ*_HO5_ values reported [3] for 2-hydroxy-7-methyl juglone (**3**), 12.23 ppm, and 3-hydroxy-7-methyl juglone (**8**), 10.99 ppm. If a mixture of **3** and **8** was present, then the observed intermediate value may be a consequence of intermolecular proton exchange. Of note, exchange can be appreciable on the NMR timescale at the observational frequency of 200 MHz used by Mahapatra et al. [2]. A mixture of **3** and **8** would also exhibit a depressed m.p., and indeed a depressed m.p. of 10°C below that of the lower melting point isomer **3** was in fact reported [2] by them. Since there are insufficient differences in the calculated *δ*_H_s of the nonlabile protons to decisively affect isomer identification of the pure compounds with only one isomer on hand, one cannot conclude from the reported [2] *δ*_H_s which compound(s) was present in the sample. Indeed, for a mixture, differences might only be seen with decent resolution which could easily be lost by even slightly poor *B*_0_ field homogeneity and which would be problematic in any case at 200 MHz. Mahapatra et al. [2] only described H-6 and H-8 as singlets, whilst we observed multiplets for the analogous protons in 7-methyl juglone (**4**). Furthermore, they did not do more to determine which compound they had or even if they had a mixture of compounds (e.g., IR and HRMS are insufficient for this purpose in the current case). Therefore, although we cannot unequivocally confirm that Mahapatra et al. [2] have the wrong structure(s) for the compound they claim as 2-hydroxy-7-methyl juglone, we must conclude that they had, most probably, a mixture of **3** and **8**.

The DFT-calculated ^13^C NMR chemical shifts are presented in Table 3 wherein it can be seen that for each isomeric pair, four carbons are able to provide substantial differentiation for assignment purposes. There is clearly no problem in identifying our 3-methoxy juglone (**6**) as the correct structure based on comparison of the calculated *δ*_C_s for 2-methoxy juglone (**12**) and **6**, in particular, *δ*_C1_, *δ*_C4_, and *δ*_C7_. Even assignment is not necessary as *δ*_C6_, *δ*_C7_, and *δ*_C8_ are all well dispersed from one another so one only needs to look for chemical shift differences (Δ*δ*) in the appropriate region. Of special note though is the Δ*δ* between *δ*_C1_ and *δ*_C4_, which has been calculated as 11.02 ppm for **12** whilst for **6** it is 2.65 ppm the latter comparing rather well to the observed value of 0.99 ppm. At the very least, ^13^C NMR acquisition, if not affording isomer identification (correlation spectra notwithstanding) outright, readily alludes to the presence of isomers in a sample and should have been the method of choice in the study of Mahapatra et al. [2].

## 3. Conclusions

The treatment of 5-hydroxy-1,4-naphthoquinone (**5**, juglone) with acetic anhydride under acidic conditions can lead to variable results as the course of the reaction is very dependent on the exact conditions applied. Herein the treatment of juglone (**5**) with acetic anhydride and H_2_SO_4_ and then subsequent treatment with methanolic HCl led to the isomeric compounds 5-hydroxy-3-methoxy-1,4-naphthoquinone (**6**, 3-methoxy juglone) and 8-hydroxy-4-methoxy-1,2-naphthoquinone (**7**) being obtained instead of the desired 2-hydroxy juglone (**2**). To correctly identify the products of a reaction in which regiospecificity may be variable or lacking altogether, it is important that appropriate analytical methods be applied, for example, ^13^C NMR, correlation spectra, or molecular modeling, to properly identify isomers or at least ascertain the presence of other isomers in the sample. In the case of comparison to the literature data, care needs to be taken that conditions are compliant with the measurements being undertaken to ensure correct interpretation of the results. Certainly, reliance on more than just one *δ*_H_ value for identification should be exercised for the dangers of not doing so are self-evident.

## 4. Experimental

### 4.1. NMR

NMR spectra were acquired using a Bruker Avance NMR spectrometer equipped with either a 5 mm inverse or a 5 mm normal configuration probe, both with z-gradient capability, at a field strength of 11.75 T operating at 500 and 125 MHz for ^1^H and ^13^C nuclei, respectively, at 25°C with samples contained in CDCl_3_. The chemical shifts of ^1^H and ^13^C nuclei are reported relative to TMS incorporated as an internal standard (*δ* = 0 ppm for both ^1^H and ^13^C). General NMR experimental details for 1D ^1^H, ^13^C, and DEPT and standard gradient-selected 2D DQF-COSY, HSQC, and HMBC spectra have been previously described [17–20] for routine structural determinations and chemical shift assignments. For 1D selective COSY (set on a *J* value of 7 Hz for vicinal coupling whilst for long-range *J*, values varied in the range 0.25–1 Hz) and NOESY [21, 22] (mixing times: 0.5, 0.75 s) experiments, selective excitation was effected using a 180° 50 ms Gaussian-shaped pulse via excitation sculpting [23]. Spin analysis was performed using the Perch iteration software [12, 13] for the extraction of *δ*_H_ and *J*_H,H _.

### 4.2. Molecular Modeling

DFT quantum chemical calculations were performed using *Gaussian 09* [24] (version A.01) and analyzed using *GaussView* (version 3.07). Geometry optimization of the structures in the gas phase was performed using the M06-2X hybrid metadensity functional [25, 26] with the 6-31G(d) basis set in tandem with vibrational analysis and thermochemistry calculations at the same level of theory. Vibrational analyses, invoking the keyword *freq = noraman*, were conducted to confirm that optimized structures were true minima on the potential energy surface by not providing imaginary frequencies and to obtain the thermodynamic contributions at 298.15 K and 1 atm wherein frequencies were left unscaled. If necessitated by the presence of imaginary frequencies, structures were re-optimized (together also with vibrational analyses and thermochemistry calculations) using tight convergence criteria by invoking the keywords *opt = tight* and *int = ultrafine*.

Absolute chemical shieldings were calculated for geometry-optimized structures in the gas phase using the GIAO method [27] and the B3LYP functional [28, 29] with the cc-pVTZ basis set. Chemical shifts were determined by subtracting calculated shieldings from the calculated shieldings of the reference compound TMS for which both *δ*_H_ and *δ*_C_ = 0.00 ppm. The chemical shifts of ^1^H and ^13^C nuclei were calibrated following literature methodology [17] using the following relationships:

(1) $$\begin{array}{c} \delta_{\text{H}}=0.9736\times \delta_{\text{calc}}+0.058\;\left(R^{2}=0.9970\right),\\ \delta_{\text{C}}=0.9880\times \delta_{\text{calc}}-3.780\;\left(R^{2}=0.9987\right). \end{array}$$

### 4.3. Synthetic Preparation of 3-Methoxy Juglone **(6)** and 8-Hydroxy-4-methoxy-1,2-naphthoquinone **(7)**

Following the method of Mahapatra et al. [2], a mixture of juglone (0.6 mmol), acetic acid anhydride (3 mL), and concentrated H_2_SO_4_ (0.1 mL) was kept overnight and then poured onto ice. The reaction mixture was extracted with chloroform, dried with MgSO_4_, and then filtered. The residue obtained after removal of the solvent in vacuo was crystallized from chloroform-hexane and the solid material collected by filtration, taken up in methanolic HCl (2 M), and then refluxed for 1 hour. After extraction of the compound using diethyl ether, column chromatography over silica gel (CHCl_3_–CH_3_OH, 95 : 5) resulted in early fractions containing **6** followed by later fractions containing **7**. Analysis of the reaction products was accomplished by HPLC (SunFire C18 3.5 **μ**m, 2.1 × 150 mm) equipped with a UV-Vis DAD and using a gradient of 15–100% acetonitrile in water with 0.1% formic acid whereby **7** was found to elute before **6**. Both compounds were subjected to semipreparative HPLC (SunFire Prep C18 5 **μ**m, 10 × 250 mm) for final purification using the same gradient of 15–100% acetonitrile in water with 0.1% formic acid. UV-Vis: for **6**, *λ*_max⁡_ 281, 411 nm; for **7**, *λ*_max⁡_ 286, 422 nm. For ^1^H and ^13^C NMR data of **6** and **7**, see Table 1.

## Figures and Tables

**Table 1:** ^
1^H and ^13^C NMR data for 3-methoxy juglone (**6**) and 8-hydroxy-4-methoxy-1,2-naphthoquinone (**7**) in CDCl_3_ at 25°C.

	3-Methoxy juglone (**6**)				8-Hydroxy-4-methoxy-1,2-naphthoquinone (**7**)			
Pos.	*δ* ^a^/ppm				*δ* ^a^/ppm			
	^ 13^C	^ 1^H^b^	Multiplicity	*J*(^1^H,^1^H)^b,c^/Hz	^ 13^C	^ 1^H^b^	Multiplicity	*J*(^1^H,^1^H)^b,d^/Hz
1	183.95	—			168.15	—		
2	110.50	6.160	qt	−0.41 (O-Me)	103.00	5.955	s	0.35 (O-Me), 0.23 (H-8), 0.18 (H-6)
3	160.08	—			179.06	—		
4	184.94	—			182.97	—		
4a	114.28	—			113.87	—		
5	161.98	11.750	dist. d	0.43 (H-7), 0.03 (H-6)	164.87	12.057	d	0.48 (H-7)
6	123.87	7.246	ho m	8.48 (H-7), 1.09 (H-8), 0.03 (HO-5)	122.43	7.138	ddd	8.61 (H-7), 1.01 (H-8), 0.18 (H-2)
7	137.20	7.640	ho m	8.48 (H-6), 7.45 (H-8), 0.43 (HO-5)	138.14	7.588	ddd	8.61 (H-6), 7.54 (H-8), 0.48 (HO-5)
8	118.95	7.630	ho m	7.45 (H-7), 1.09 (H-6)	117.51	7.411	ddd	7.54 (H-7), 1.01 (H-6), 0.23 (H-2)
8a	132.06	—			131.81	—		
CH_3_O	56.61	3.920	d	−0.41 (H-2)	56.83	3.994	d	0.35 (H-2)

d: doublet; dist.: distorted; ho: higher order; m: multiplet; qt: quartet; s: singlet. ^a1^H and ^13^C spectra referenced internally to TMS (*δ* = 0.00 ppm for both nuclei). ^b^Chemical shifts and coupling constants extracted using the Perch simulation software [12, 13]. ^c^Couplings in the aromatic ring were determined to be positive from higher-order analysis and were consistent with signs from DFT calculations except for _ _^4^ *J*_H6,HO5_ which was also found to be positive by spin simulation. Despite its small value, _ _^4^ *J*_H6,HO5_ was evident in the lineshape. _ _^5^ *J*_H2,OMe_ determined to be negative by DFT calculations. ^d^_ _^4^ *J*_H6,H8_ and _ _^5^ *J*_HO5,H7_ were set positive in analogy with **6** and were consistent with signs from DFT calculations. Signs of _ _^5^ *J*_H2,H8_, _ _^5^ *J*_H2,OMe_, and _ _^7^ *J*_H2,H6_ assigned by DFT calculations.

**Table 2:** DFT-calculated ^1^H NMR *δ*_H_s (ppm) for compounds **2**, **3**, **6**, **8**, **11**, and **12**.

Compound	H-2/HO-2/CH_3_O-2	H-3/HO-3/CH_3_O-3	HO-5	H-6	H-7/CH_3_-7	H-8
2-Hydroxy juglone (**2**)	7.62	6.06	12.33	7.26	7.45	7.76
3-Hydroxy juglone (**11**)	6.09	7.42	11.04	7.11	7.59	7.77
2-Hydroxy-7-methyl juglone (**3**)	7.57	6.03	12.34	7.05	2.38	7.62
3-Hydroxy-7-methyl juglone (**8**)	6.05	7.44	11.05	6.91	2.40	7.62
**3,** observed [2]	no	6.08	11.69	7.01	2.41	7.42
2-Methoxy juglone (**12**)	3.68	5.78	12.19	7.17	7.45	7.72
3-Methoxy juglone (**6**)	5.80	3.69	11.76	7.11	7.51	7.73
**6**, observed (this work)	6.16	3.92	11.75	7.25	7.64	7.63

Legend: no: not observed.

**Table 3:** DFT-calculated ^13^C NMR chemical shifts (ppm) for compounds **2**, **3**, **6**, **8**, **11**, and **12**.

Compound	C-1	C-2	C-3	C-4	C-4a	C-5	C-6	C-7	C-8	C-8a	CH_3_
2-Hydroxy juglone (**2**)	181.93	156.99	107.41	190.08	112.87	163.49	127.36	133.75	118.07	129.19	—
3-Hydroxy juglone (**11**)	181.77	108.35	156.87	185.51	111.33	163.53	121.69	138.31	118.51	132.99	—
2-Hydroxy-7-methyl juglone (**3**)	182.16	156.97	107.50	189.51	110.56	163.81	126.85	147.59	118.88	128.95	19.63
3-Hydroxy-7-methyl juglone (**8**)	182.07	108.00	157.30	184.36	109.37	163.93	121.10	152.92	119.59	133.35	20.78
2-Methoxy juglone (**12**)	178.88	162.04	106.98	189.90	112.93	163.45	124.69	134.41	117.97	131.52	51.86
3-Methoxy juglone (**6**)	181.91	107.32	161.80	184.56	112.91	163.95	122.57	136.40	117.42	131.94	51.74
**6**, observed (this work)	183.95	110.50	160.08	184.94	114.28	161.98	123.87	137.20	118.95	132.06	56.61
